# Two-Dimensional Near-Atom-Thickness Materials for Emerging Neuromorphic Devices and Applications

**DOI:** 10.1016/j.isci.2025.112255

**Published:** 2025-03-21

**Authors:** Tae-Jun Ko, Hao Li, Sohrab Alex Mofid, Changhyeon Yoo, Emmanuel Okogbue, Sang Sub Han, Mashiyat Sumaiya Shawkat, Adithi Krishnaprasad, Molla Manjurul Islam, Durjoy Dev, Yongjoon Shin, Kyu Hwan Oh, Gwan-Hyoung Lee, Tania Roy, Yeonwoong Jung

## Main text

(iScience *23*, 101676; November 20, 2020)

In the originally published article, author Yongjoon Shin's name appeared as Yongjun Shin. The correct spelling should be Yongjoon Shin. The authors apologize for any confusion.

